# Impact of Sodium–Glucose Cotransporter 2 (SGLT2) Inhibitors on Arterial Stiffness and Vascular Aging—What Do We Know So Far? (A Narrative Review)

**DOI:** 10.3390/life12060803

**Published:** 2022-05-27

**Authors:** Cristina Andreea Adam, Razvan Anghel, Dragos Traian Marius Marcu, Ovidiu Mitu, Mihai Roca, Florin Mitu

**Affiliations:** 1 Clinical Rehabilitation Hospital, Cardiovascular Rehabilitation Clinic, Pantelimon Halipa Street nr. 14, 700661 Iaşi, Romania; cristina-adam@email.umfiasi.ro (C.A.A.); razvan-constantin-anghel@email.umfiasi.ro (R.A.); mihai.c.roca@umfiasi.ro (M.R.); florin.mitu@umfiasi.ro (F.M.); 2 Department of Internal Medicine, University of Medicine and Pharmacy, Grigore T. Popa, University Street nr. 16, 700115 Iaşi, Romania; 3 Sf. Spiridon Clinical Emergency Hospital, Independence Boulevard nr. 1, 700111 Iasi, Romania

**Keywords:** vascular aging, arterial stiffness, SGLT2 inhibitors, early vascular aging, diabetes mellitus, cardiovascular risks

## Abstract

Vascular aging, early vascular aging or supernormal vascular aging are concepts used for estimating the cardiovascular risk at a certain age. From the famous line of Thomas Sydenham that “a man is as old as his arteries” to the present day, clinical studies in the field of molecular biology of the vasculature have demonstrated the active role of vascular endothelium in the onset of cardiovascular diseases. Arterial stiffness is an important cardiovascular risk factor associated with the occurrence of cardiovascular events and a high risk of morbidity and mortality, especially in the presence of diabetes. Sodium–glucose cotransporter 2 inhibitors decrease arterial stiffness and vascular resistance by decreasing endothelial cell activation, stimulating direct vasorelaxation and ameliorating endothelial dysfunction or expression of pro-atherogenic cells and molecules.

## 1. From Vascular Aging to Arterial Stiffness

Vascular aging affects large elastic arteries, the main changes identified in the vascular wall being luminal enlargement with wall thickening and a reduction in elastic properties also known as stiffening [1]. From the famous line of Thomas Sydenham that *“a man is as old as his arteries”* to the present day, clinical studies conducted in the field of molecular biology of the vasculature have demonstrated the active role of the vascular endothelium in maintaining general homeostasis, as well as in the onset and progression of CVD [2,3,4]. With advancing age, the amount of elastin in the central arteries decreases, leading to fiber fatigue and fracture, which contributes to arterial aging, along with vascular calcification and endothelial dysfunction [5]. This article aims to review the beneficial effects of sodium–glucose cotransporter 2 (SGLT2) inhibitors on vascular function, which has been little studied so far but has promising results in in vitro studies.

The relationship between arterial ageing and arterial stiffness is bidirectional. Hypertension, diabetes mellitus, dyslipidemia or obesity are cardiovascular risk factors that accelerate the process of arterial stiffening and ageing. Additionally, arterial stiffening per se may be considered an independent risk factor in the development of atherosclerosis and, consequently, lead to various cardiovascular diseases (CVD) [6,7,8]. Arterial stiffness leads to increased pulse wave velocity and pulse pressure, which are markers of the pulsatile energy content of the pressure waveform. An increase in this leads to additional stress on the heart [9]. There is a directly proportional relationship between pulse pressure and the risk of cardiovascular disease, with increased pulse pressure associated with an increased risk of myocardial infarction, heart failure, arrhythmias and stroke [10,11,12,13].

**Figure 1:**
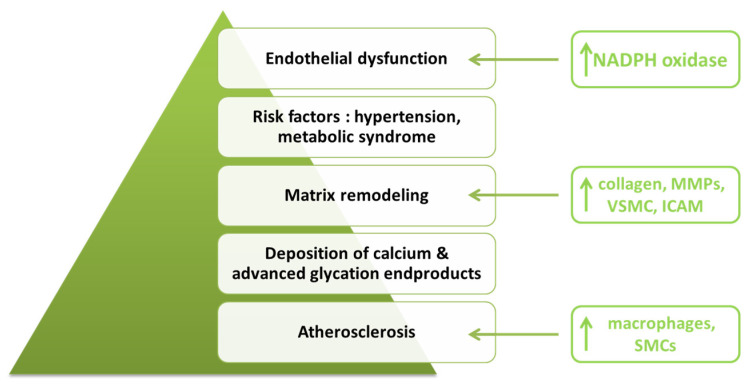
Factors contributing to arterial aging (adapted after [1]). (NADPH: nicotinamide adenine dinucleotide phosphate oxidase; MMPs: matrix metalloproteinases; VSMC: vascular smooth muscle cells; ICAM: intercellular adhesion molecule; SMCs: smooth muscle cells).

Medial degeneration is the key factor that causes arterial stiffness [14]. The structure of the media, which is made up mainly of collagen fibers, elastin and vascular smooth muscle cells (VSMC), degenerates, causing muscle fiber rupture over time [15]. In certain pathological situations (such as in hypertension or atherosclerosis), proteins in the cellular matrix structure undergo proteolysis, generating bioactive fragments with an active role in vascular remodeling processes [4,16]. Extracellular matrix molecules (elastin, collagens, proteoglycans or glycoproteins) synthesized during the growth period ensure tissue homeostasis [17]. Elastic fibers are considered to be the strongest structures, with an estimated durability of about 40 years [18]. In the adult period, there is a decrease in their activity with a very low turnover rate for elastin and collagen fibers, leading to the disruption of homeostasis and the appearance of morphological and functional disorders exacerbated in the context of the presence of related pathologies such as CVD [19] (Figure 1). Their degradation occurs naturally with advancing age or as a result of excessive mechanical stress that stimulates the appearance of a permanent inflammatory environment, which stimulates vascular calcification processes [20]. Besides mechanical stress, telomere dysfunction, oncogene activation or deoxyribonucleic acid (DNA) damage contribute to cellular senescence, exacerbating the arterial aging process [21]. Vascular fibrosis is characterized by the accumulation of extracellular matrix, especially collagen fibers, contributing to the remodeling process in hypertension, restenosis or atherosclerosis [22,23,24,25].

The deposition of calcium and advanced glycation end products reduce arterial distensibility, particularly after the 5th decade [26]. Calcification occurs predominantly in the intimal plaques and the medial elastic fiber network [26,27]. The calcification of medial elastic fibers, also known as elastocalcinosis, occurs independently of the calcification of atheromas [28]. Medial elastocalcinosis mainly affects elastic fibers [29], and over time, their fragmentation has been observed in the coronary arteries [30]. Peripheral artery calcification is associated with an increased risk of limb acute events in patients with peripheral artery disease [31,32,33]. Zettervall et al. [31] demonstrated significant correlations between the risk of ischemia and age, diabetes duration, hypertension, the artery occlusion score and peripheral artery calcification. The identification of potential biomarkers associated with vascular calcification, as well as the understanding of the molecular mechanisms underlying pathophysiological changes, are research directions that have been increasingly addressed in recent years [34,35]. Chen et al. [34] compared the genes of subjects with normal arteries to those of patients with significant vascular changes and demonstrated the presence of genetic changes associated with the development and progression of vascular calcification. They identified genes with a significantly high expression in vascular calcification, which can be seen both as potential biomarkers and future therapeutic targets. 

Peripheral resistance associated with endothelial dysfunction occurs as a consequence of altered motor function with advancing age. Simultaneously, there is a disturbance of the balance between antioxidant status and oxidative stress [36,37]. Tobacco smoking, one of the most common cardiovascular risk factors, causes the appearance of oxygen free radicals, thus further contributing to the accentuation of oxidative stress [38]. Aging is considered a major determinant of CVD-related events [37], altered mitochondrial functions and oxidative stress (apoptosis, bioenergetics or inflammation) being factors associated with the occurrence of heart failure, cardiac hypertrophy or diabetic cardiomyopathy [39,40,41]. Oxidative stress promotes vascular inflammation even in the absence of risk factors associated with atherogenesis via reactive oxygen species [42,43,44]. 

Arterial stiffness is an important cardiovascular risk factor associated with the occurrence of cardiovascular events and a high risk of morbidity and mortality [45,46]. Pulse pressure and mean arterial pressure are its main determinants, themselves having both prognostic and therapeutic value for predicting CVD-associated risk in patients with diabetes mellitus [47,48,49]. Mean arterial pressure and pulse pressure have a proven beneficial role in assessing prognosis, the risk of hospitalization for heart failure (*p* < 0.002) or that of all-causes mortality (*p* < 0.007) in heart failure patients with preserved ejection fraction [48,50,51]. Arterial stiffness accelerates the onset and progression of neuropathy, nephropathy and retinopathy in patients with type 2 diabetes mellitus [52,53,54,55]. 

## 2. Effects of SGLT2 Inhibitors on Vascular Aging

### 2.1. Cardiorenal Benefits of SGLT2 Inhibitors

**Figure 2:**
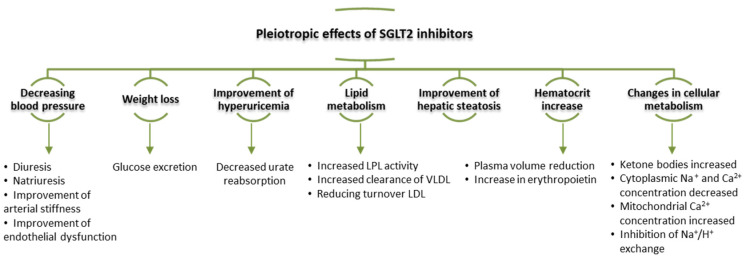
Pleiotropic effects of SGLT2 inhibitors (adapted after [69]) (LPL: lipoprotein lipase; VLDL: very low density lipoprotein; LDL: low density lipoprotein).

Renal glucose reabsorption is crucial in maintaining adequate glycemic control. Clinical studies have identified three families of glucose transporters in humans: glucose facilitators, sodium-glucose cotransporter and sugars that will eventually be exported transporters (SWEETs) [56,57,58]. Among these, sodium-glucose cotransporter mediates most of the glucose reabsorption process in the early proximal tubule via an insulin-independent mechanism [59]. By reducing hyperglycemia, SGLT2 inhibitors also reduce glucotoxicity in both kidney and extrarenal organs [60,61,62]. The pleiotropic effects such as reducing blood pressure, weight loss or the inhibition of hyperuricemia contribute to cardiovascular protection [60,63] (Figure 2). SGLT2 inhibitors’ contribution to erythropoietin production, extracellular volume homeostasis or interaction with the angiotensin system and the renal sympathetic nervous system were also investigated [63]. 

Until the development of SGLT2 inhibitors, glucose-lowering agents had little or no impact on CVD progression in patients with diabetes mellitus [64]. Of all the agents in this class, dapagliflozin is the only one approved for the treatment of patients with type 1 diabetes in the European Union to date [64]. Fibroblasts in the interstitial tubules are responsible for the synthesis of erythropoietin, the production of which is mediated by the hypoxia-inducible factor in the presence of oxygen [65,66]. In patients with diabetes mellitus, tubulointerstitial hypoxia occurs as a result of excessive oxygen consumption in the process of impaired glucose reabsorption [67]. SGLT2 inhibitors relieve hypoxia in the interstitial tubules by decreasing workload at this level, thereby improving erythropoietin production in fibroblasts. Clinical studies in the literature report an average increase in hematocrit value of 2–4% compared to a placebo [68]. Hematocrit changes are influenced by the degree of renal dysfunction in patients treated with empagliflozin. In this regard, Sano et al. [68] emphasize the beneficial effect of administering this SGLT2 inhibitor to diabetic patients with chronic kidney disease stages 2 and 3, with no beneficial effects observed in patients with severe renal impairment.

**Figure 3:**
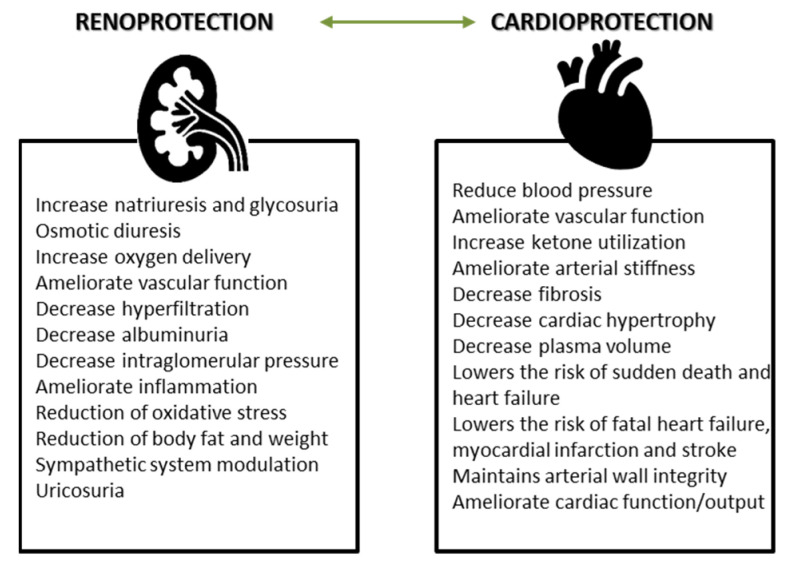
Cardioprotective and renoprotective effects of SGLT2 inhibitors (adapted after [100,102]).

Phlorizin was the first SGLT2 inhibitor isolated in 1835 from the bark of an apple tree by a group of French chemists [70,71]. Decades later, Kahn et al. [72] demonstrated in a diabetic rat model that phlorizin causes the decrease in both fasting and postprandial blood glucose levels, independent of insulin secretion. Chasis et al. [73] concluded that the administration of phlorizin leads to lower blood pressure and lower body weight and increases renal glucose excretion, but its use as an oral antidiabetic is not possible due to rapid lactase–phlorizin hydrolase-induced degradation and reduced absorption in the gastrointestinal tract [74]. The protective role in cardiovascular, renal and vascular function modulation is a class one for SGLT2 inhibitors (Figure 3), but in the case of phlorizin, it could only be demonstrated in clinical studies on animals. Shen et al. [75] investigated the effect of phlorizin on vascular complications in an animal model. At a 10-week follow-up, they concluded that its administration to diabetic mice resulted in decreased blood glucose and advanced glycation end products (AGEs) concentration, thus conferring a preventive role for the occurrence and progression of macrovascular complications. In a similar study, Pei et al. [76] emphasized the protective role of phlorizin in preventing the development of diabetic nephropathy by modulating metabolic processes involved in protein and lipid transport and metabolism.

SGLT2 inhibitors are associated with favorable cardiovascular and kidney outcomes in people with or without diabetes mellitus [77]. The main benefits are a reduction in the risk of cardiovascular death and hospitalization for heart failure, regardless of the presence of diabetes or heart failure [69]. Heart failure is one of the most commonly encountered CVD and is considered a public health problem in terms of economic, social and associated medical management implications [78,79,80]. In the context of the ongoing industrialization of society and the increasing prevalence of cardiovascular risk factors and hence cardiovascular damage, the development and introduction of new therapeutic molecules for patients with heart failure leads to a decrease in the risk of morbidity and mortality [59,81,82]. EMPA-REG OUTCOME (Empagliflozin Cardiovascular Outcomes Event Trial in Type 2 Diabetes Mellitus Patients—Removing Excess Glucose) investigators [83] concluded that diabetic patients with important cardiovascular risk factors had an early reduction in both cardiovascular and renal events following treatment with empagliflozin. Similar results have been reported by other clinical trials such as CANVAS (Canagliflozin Cardiovascular Assessment Study) [84] or CREDENCE (Canagliflozin and Renal Outcomes in Type 2 Diabetes and Nephropathy) [85] for canagliflozin and DECLARE-TIMI 58 (Dapagliflozin and Cardiovascular Outcomes in Type 2 Diabetes) [86] for dapagliflozin. In addition to the trials mentioned above, other clinical trials demonstrating the cardiovascular protective role by reducing the hospitalization rate in heart failure or optimizing the treatment of these patients (DAPA-HF, DELIVER HFpEF, EMPEROR-Preserved, EMPEROR-Reduced) are cited in the literature [87]. The renal protective role conferred by SGLT2 inhibitors is to reduce the decline in renal function (proven outcome in the CANVAS Program, DECLARE-TIMI 58 and EMPA-REG OUTCOME trials) or to stabilize and slow the progression of chronic kidney disease in previously diagnosed patients (EMPA-Kidney, DAPA-CKD) [87]. Many of the clinical trials reviewed included patients with an estimated glomerular filtration rate (eGFR) of less than 60 mL/min/1.73 m^2^ [88]. A meta-analysis conducted by Lo et al. [89] concluded that cardiorenal protection is maintained in those with renal impairment below the mentioned threshold. The molecular mechanisms in relation to SGLT2 inhibitors involved in modulating the processes of arterial stiffening and vascular aging are complex, currently representing a major interest of the scientific community. Several clinical studies in the field have emphasized the beneficial role of Na^+^ homeostasis in cardioprotection and the modulation of vascular function [90]. Thus, the inhibition of essential proteins in the cellular metabolism of Na^+^ as well as cardiac Na^+^/H^+^ exchanger 1 [91,92,93], Ca^2+^/calmodulin-dependent protein kinase II [94] and late Na^+^ current helps prevent the progression of heart failure [90,95]. In addition to modulating processes involved in Na metabolism, the inappropriate activation of the RAS system, as well as increased sympathetic nervous system activity are processes antagonized by SGLT2 inhibitors in in vitro and in vivo clinical studies [96]. 

The pathophysiology of chronic heart failure involves various signaling pathways that are not localized to cardiac myocytes, with consecutive involvement of numerous cells such as fibroblasts, vascular cells, cytokines or chemokines [97] that mediate complex processes, stimulating the continuous development of bio-molecular analyzing techniques [98]. 

SGLT2 inhibitors can be used for both primary and secondary prevention population, the cardiorenal benefits being variable depending on the presence or absence of established CVD [99]. Thus, in diabetic patients with multiple cardiovascular risk factors, SGLT2 inhibitors provide renal protection and decrease the risk of hospitalization due to heart failure but do not decrease the risk of major cardiovascular events, as seen in patients with established CVD [77,100,101]. In a meta-analysis based on the aforementioned clinical trials, Zelniker et al. [101] concluded the beneficial role of SGLT2 inhibitors in decreasing the risk of major cardiovascular events by 11% (*p* = 0.0014) in patients with atherosclerotic disease than in those with cardiovascular risk factors (*p* = 0.0501) associated with myocardial infarction (by 11%, *p* = 0.0177) and cardiovascular death (by 16%, *p* = 0.0023). 

### 2.2. SGLT2 Inhibitors Modulate Vascular Function

**Figure 4:**
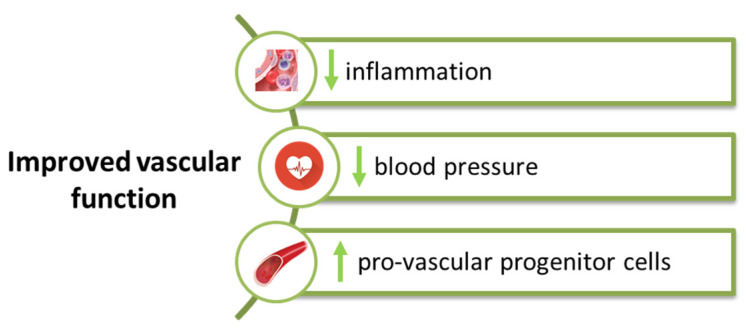
Effect of SGLT2 inhibitors at the vascular level.

The vascular endothelium is the site of multiple pathophysiological processes in which the action of pro-inflammatory mediators induces endothelial dysfunction, vascular inflammation or vascular remodeling in all segments of the vessel wall [103,104,105]. The effects of SGLT2 inhibitors are also reflected in the vascular function by modulating blood pressure, reducing inflammatory status or inhibiting the sympathetic nervous system [69]. Decreasing oxidative stress and increasing circulating pro-vascular progenitor cells lead to improved vascular function (Figure 4). 

SGLT2 inhibitors decrease arterial stiffness and vascular resistance by decreasing endothelial cell activation, stimulating direct vasorelaxation and ameliorating endothelial dysfunction or the expression of pro-atherogenic cells and molecules [69]. Heart failure is characterized by a reduced degree of chronic systemic inflammation, which leads to endothelial dysfunction. Uthman et al. [92] concluded that both empagliflozin and dapagliflozin modulate nitric oxide bioavailability by inhibiting the formation of reactive oxygen species and not by decreasing the expression of endothelial nitric oxide synthase (eNOS) or eNOS-modulated pathways at the vascular level. SGLT2 inhibitors mediate the restoration of provascular progenitor cells in patients with type 2 diabetes mellitus [69,106]. Hess et al. [107] emphasized the beneficial effect of empagliflozin in decreasing the number of pro-inflammatory M1 cells and increasing the M2 ones, with anti-inflammatory action [108].

SGLT2 inhibitors ameliorate arterial stiffness and vascular resistance by reducing blood pressure, a mechanism demonstrated in multiple clinical studies to date [109,110]. Chilton et al. [111] conducted a post hoc analysis on several trials in which patients with type 2 diabetes treated with empagliflozin were enrolled. Besides the reduction in both systolic and diastolic blood pressure (*p* < 0.001), in the pulse pressure (−2.3 mmHg) and mean arterial pressure values (−2.1 mmHg), the follow-up results showed a decrease in ambulatory arterial stiffness index values, signifying a reduction in arterial stiffness (*p* = 0.059 vs. placebo). Similar results were obtained with the administration of canagliflozin, its administration in patients with type 2 diabetes mellitus leading to an improvement in blood pressure values and arterial stiffness determinants [45,112], having thus a beneficial effect on vascular function.

### 2.3. SGLT2 Inhibitors and Vascular Aging—In Vitro Cell and Animal Evidence

To date, multiple in vivo and in vitro studies have been performed, demonstrating the anti-inflammatory role of SGLT2 inhibitors in coronary microvascular endothelial cells, heart or kidney. Dapagliflozin decreases the rate of inflammasome activation, thus having an anti-inflammatory and anti-fibrotic effect, independently of lowering glucose values in type 2 diabetic mice [113]. The anti-inflammatory role of dapagliflozin was also demonstrated by Gaspari et al. [114] in an in vitro study on human vascular endothelial cells stimulated with TNF-α or hyperglycemic conditions, who observed a decrease in the expression of intercellular adhesion molecule-1, vascular cell adhesion molecule-1, plasminogen activator inhibitor type 1 and (nuclear factor kappa B) NFκB expression. They also highlighted the acute vaso-protective effects of dapagliflozin by inducing endothelium-independent vasorelaxation, as well as a decrease in the expression of vascular adhesion molecule with macrophage vessel wall infiltration as a result of chronic administration. Besides that, dapagliflozin mediates cardiac remodeling and attenuates cardiac fibrosis by decreasing myofibroblast infiltration in rats after myocardial infarction through a reactive oxygen and nitrogen species (RONS)/activator of transcription 3 (STAT3)-dependent pathway [115]. Li et al. [116] investigated the effect of dapagliflozin on thoracic aorta smooth muscle cells in rabbits and concluded that this SGLT2 inhibitor induces vasodilatation independent of the endothelium by channeling the activation of voltage-dependent K^+^ channels and protein kinase G signaling pathways.

In a recent study conducted by Hodrea et al. [64], the group of investigators demonstrated that dapagliflozin ameliorates cardiac inflammation induced by diabetes mellitus similarly to the effect observed in type 2 diabetic rodent models by Aragón-Herrera et al. [117] or Ye et al. [118]. The anti-inflammatory and anti-fibrotic effect of dapagliflozin was also evidenced in the study proposed by the last mentioned investigators, dapagliflozin administration leading to decreased inflammasome activation and decreased levels of IL-1β [118].

In a similar preclinical study, empagliflozin improved cell viability and stimulated ATP replenishment in endothelial cells previously exposed to hypoxia-reoxygenation injury [92,119]. Lin et al. [120] highlighted the beneficial effect of empagliflozin in ameliorating cardiovascular injury, preventing cardiac remodeling and ameliorating vascular dysfunction or cognitive decline in obese and type 2 diabetic mice, raising the hypothesis of its administration in cases with diabetic macrovascular disease and associated cognitive decline. Empagliflozin has a major impact on microvascular complications associated with diabetes mellitus. It preserves the microvascular barrier integrity and associated homeostasis through the enhancement of eNOS phosphorylation and vasorelaxation endothelium-dependent, which leads to an improvement in microvessel density and perfusion [121,122,123]. Based on the idea that hyperglycemia contributes to arterial stiffness, Aroor et al. [124] studied the effects of empagliflozin administration in type 2 diabetic female mice and concluded that its administration is associated with an improvement in the renal microvasculature stiffening measured using the renal resistivity index (*p* = 0.003). These findings are supported by pre-existing data in the literature that increases in the degree of cortical tissue periarterial and tubulointerstitial fibrosis, alongside aortic stiffness, lead to renal vascular stiffness and albuminuria in patients with diabetes mellitus [125,126]. Aragón-Herrera et al. [117] demonstrated the cardioprotective role of empagliflozin by ameliorating cardiac metabolome and lipidome through regulation of cellular energy homeostasis AMP-activated protein kinase and stimulating autophagy at the cardiac level while decreasing the cardiac mRNA levels of pro-inflammatory molecules. 

Empagliflozin, dapagliflozin and canagliflozin inhibit in vitro the progression of arterial atherosclerotic lesions related to vascular aging by blocking cell proliferation and migration of VSMC. The effect of canagliflozin is dose-dependent, but in the case of the other two representatives, it has been demonstrated that an extremely high concentration is required for a moderate decrease in VSMC processes [127,128]. In vitro administration of canagliflozin is also associated with the reduction in pro-inflammatory agents such as TNF receptor 1, IL-6, matrix metalloproteinase 7 and fibronectin 1 [129,130]. 

Most clinical studies documented canagliflozin as the only SGLT2 inhibitor that activates AMP-activated protein kinase (AMPK) [131] and inhibits pro-inflammatory status by decreasing the concentration of chemokines and cytokines without interfering with IL-1β signaling pathways [132]. In a recent study, Cai et al. [133] emphasized the protective role of empagliflozin in mitochondrial metabolism through the activation of the AMPKα1/UNC-51-like kinase (ULK1)/FUN14 domain containing 1 (FUNDC1)/mitophagy pathway both in intro and in vivo [134,135,136]. In addition to acting at the level of mitochondrial metabolism, empagliflozin alleviates microvascular dysfunction by mediating the interaction between cardiac microvascular endothelial cells and cardiac myocytes [137,138]. In a similar study, Soares et al. [139] concluded that empagliflozin reduces arterial stiffness in aged mice by increasing the activation of eNOS and downregulating pathways involved in the synthesis of reactive oxygen species. 

Ipragliflozin is another representative of the SGLT2 inhibitor class, less known than the main representatives presented above, but with a similar effect on endothelial function proven in multiple in vitro studies [52]. Salim et al. [140] conducted a study on the impact of ipragliflozin on endothelial cells in diabetic mice and demonstrated its active role in preventing endothelial dysfunction by decreasing the expression of reactive oxygen species or pro-inflammatory molecules such as monocyte chemotactic protein-1 (MCP-1), vascular cell adhesion molecule-1 (VCAM-1) and intercellular adhesion molecule-1 (ICAM-1). 

### 2.4. SGLT2 Inhibitors and Vascular Aging—Clinical Evidence

SGLT2 inhibitors have been shown to be beneficial in numerous clinical trials involving patients with type 2 diabetes [52]. Santiago et al. [141] investigated the effect of dapagliflozin on arterial stiffness by measuring the velocity of the carotid-femoral pulse at 6 and 12 months from enrollment in 32 diabetic patients. At the follow-up, significant decreases in the velocity of the carotid-femoral pulse were observed, but without significant correlations between these values and blood pressure, blood glucose or weight. In a similar study, Bosch et al. [142] demonstrated an improvement in vascular parameters (central systolic blood pressure, central pulse pressure and reflected wave amplitude, all with statistically significant value) associated with arterial stiffness secondary to empagliflozin administration due to its combined anti-inflammatory mechanism.

The concomitant administration of empagliflozin and linagliptin compared to metformin and linagliptin resulted in decreased central blood pressure (*p* = 0.009) and pulse wave velocity (*p* = 0.043) values, suggestive of significant improvement in vascular function [143]. Another clinical trial conducted by Bekki et al. [144] emphasized that switching DPP-4 inhibitors to tofogliflozin was associated with an improvement in arterial stiffness in type 2 diabetic patients as a result of improved liver function. The cardio-ankle vascular index (CAVI) was used to assess arterial stiffness. The 6-month follow-up showed improvement in both CAVI and liver parameters (aspartate transaminase, γ-glutamyl transferase and AGEs), suggesting that serum levels of AGEs may be used to identify patients with improved vascular function after treatment with tofogliflozin. The benefic effect of tofogliflozin on arterial stiffens has been demonstrated by Katakami et al. [145], who evaluated the impact of the SGLT2 inhibitor on the brachial-ankle pulse wave velocity in diabetic patients without known CVD. Tofogliflozin decreased the progression of arterial stiffening processes compared to conventional treatment (*p* < 0.005), including after adjustment for cardiovascular risk factors. Unfortunately, the same group of investigators did not obtain the same results in the analysis of carotid lesions, with no significant changes in carotid intima-media thickness after administration of tofogliflozin [146]. 

## 3. Early Vascular Aging (EVA) vs. Supernormal Vascular Aging (SUPERNOVA)—From “Inflammaging” to SGLT2 Inhibitors 

Vascular age is a concept used for estimating the cardiovascular risk at a certain age [147]. Early vascular aging (EVA) and supernormal vascular aging are two extreme vascular ageing phenotypes. EVA or arterial stiffness is a concept mentioned in the literature for the first time in 2008 [148] and includes premature changes in the structure and function of the arteries due to the action of cardiovascular risk factors, environmental factors, genetic predisposition or fetal programming [149,150]. On the other hand, supernormal vascular aging is defined as abnormally low arterial stiffness [151]. Although arterial stiffening is a physiological consequence of aging, there are studies in the literature on subgroups that attest to the lack of age-related increases in blood pressure, atherosclerotic lesions or vascular stiffening [152,153,154]. Laurent et al. [151] defined SUPERNOVA as subjects with still elastic arteries and a large discrepancy between chronological and vascular age despite exposure to the factors mentioned before. 

Between these two entities, EVA and SUPERNOVA are patients with healthy vascular aging interpreted as low blood pressure and pulse wave velocity over 50 years old [155]. Bruno et al. [148] demonstrated that estimation of the age difference between the vascular and chronological one might be a useful clinical tool for identifying patients with SUPERNOVA phenotype. Individuals with a vascular age at least 6 years younger than their chronological age are associated with a 40% lower risk of cardiovascular events compared to subjects with a normal vascular age despite the prevalence of cardiovascular risk factors. Various pathophysiological mechanisms underlying EVA are incriminated such as shorter telomere length, modulating endothelial cell activity or nitric oxide bioavailability [156,157,158]. 

Inflammation serves as a precursor for EVA besides atherothrombosis and diabetes mellitus [159]. Vascular aging and diabetes mellitus are linked by the inflammatory status, with common risk factors, which allows the observation of an integrated approach in terms of prevention and primary interventions [160,161]. “Inflammaging” is a term usually used to describe the chronic low-degree inflammatory status associated with aging [161]. Similarly to “metaflammation” [162], the concepts behind these two terms are based on the modulation of the inflammatory process, one of the main pillars associated with both the onset and progression of diabetes and ageing [163]. Drug treatment with SGLT2 inhibitors can reduce AVA by increasing glucosuria and lowering blood pressure [164]. 

SGLT2 inhibitors, exercise training, healthy lifestyle attitudes and caloric restriction provide a protective, anti-inflammatory role that promotes normal vascular aging and also decreases the risk of CVD development and progression in patients with diabetes mellitus [165].

## 4. Conclusions

SGLT2 inhibitors are the latest class of antidiabetic medication with cardioprotective and renoprotective effects. SGLT2 inhibitors improve vascular function by increasing the bioavailability of nitric oxide in the endothelium and modulating the proliferation, migration, survival and senescence of endothelial cells. Their antioxidant and anti-inflammatory effect slow the arterial stiffening process in diabetic patients.

## References

[1] Lee H.-Y., Oh B.-H. (2010). Aging and Arterial Stiffness. Circ. J..

[2] Avolio A. (2013). Arterial Stiffness. Pulse.

[3] Najjar S.S., Scuteri A., Lakatta E.G. (2005). Arterial aging: Is it an immutable cardiovascular risk factor?. Hypertension.

[4] Greenwald S.E. (2007). Ageing of the conduit arteries. J. Pathol..

[5] Izzo J.L., Shykoff B.E. (2001). Arterial stiffness: Clinical relevance, measurement, and treatment. Rev. Cardiovasc. Med..

[6] Mitchell G. (2009). Arterial Stiffness and Wave Reflection: Biomarkers of Cardiovascular Risk. Artery Res..

[7] Mitchell G.F., van Buchem M.A., Sigurdsson S., Gotal J.D., Jonsdottir M.K., Kjartansson Ó., Garcia M., Aspelund T., Harris T.B., Gudnason V. (2011). Arterial stiffness, pressure and flow pulsatility and brain structure and function: The Age, Gene/Environment Susceptibility—Reykjavik Study. Brain.

[8] Buliga-Finis O., Ouatu A., Badescu M.C., Dima N., Tănase D., Richter P., Rezuş C. (2022). Beyond the Cardiorenal Syndrome: Pathophysiological Approaches and Biomarkers for Renal and Cardiac Crosstalk. Diagnostics.

[9] Malone A.F., Reddan D.N. (2010). Pulse Pressure. Why is it Important?. Perit. Dial. Int..

[10] Franklin S.S., Khan S.A., Wong N.D., Larson M.G., Levy D. (1999). Is pulse pressure useful in predicting risk for coronary heart Disease? The Framingham heart study. Circulation.

[11] Mitchell G.F., Vasan R.S., Keyes M.J., Parise H., Wang T.J., Larson M.G., D’Agostino R.B., Kannel W.B., Levy D., Benjamin E.J. (2007). Pulse pressure and risk of new-onset atrial fibrillation. JAMA.

[12] Amabile N., Cheng S., Renard J.M., Larson M.G., Ghorbani A., McCabe E., Griffin G., Guerin C., Ho J.E., Shaw S.Y. (2014). Association of circulating endothelial microparticles with cardiometabolic risk factors in the Framingham Heart Study. Eur. Heart J..

[13] Domanski M., Mitchell G., Pfeffer M., Neaton J.D., Norman J., Svendsen K., Grimm R., Cohen J., Stamler J. (2002). MRFIT Research Group Pulse pressure and cardiovascular disease-related mortality: Follow-up study of the Multiple Risk Factor Intervention Trial (MRFIT). JAMA.

[14] Jacob M.P. (2003). Extracellular matrix remodeling and matrix metalloproteinases in the vascular wall during aging and in pathological conditions. Biomed. Pharmacother..

[15] Pihlajaniemi T. (2013). Many dimensions of extracellular matrix research. Duodecim.

[16] Keeley F.W., Johnson D.J. (1986). The effect of developing hypertension on the synthesis and accumulation of elastin in the aorta of the rat. Biochem. Cell Biol..

[17] Jacob M.-P. (2006). Extracellular matrix and vascular ageing. Med. Sci. (Paris).

[18] Arribas S.M., Hinek A., González M.C. (2006). Elastic fibres and vascular structure in hypertension. Pharmacol. Ther..

[19] Freitas-Rodríguez S., Folgueras A.R., López-Otín C. (2017). The role of matrix metalloproteinases in aging: Tissue remodeling and beyond. Biochim. Biophys. Acta Mol. Cell Res..

[20] Ribeiro-Silva J.C., Nolasco P., Krieger J.E., Miyakawa A.A. (2021). Dynamic Crosstalk between Vascular Smooth Muscle Cells and the Aged Extracellular Matrix. Int. J. Mol. Sci..

[21] Di Micco R., Krizhanovsky V., Baker D., d’Adda di Fagagna F. (2021). Cellular senescence in ageing: From mechanisms to therapeutic opportunities. Nat. Rev. Mol. Cell Biol..

[22] Ponticos M., Smith B.D. (2014). Extracellular matrix synthesis in vascular disease: Hypertension, and atherosclerosis. J. Biomed. Res..

[23] Osherov A.B., Gotha L., Cheema A.N., Qiang B., Strauss B.H. (2011). Proteins mediating collagen biosynthesis and accumulation in arterial repair: Novel targets for anti-restenosis therapy. Cardiovasc. Res..

[24] Intengan H.D., Schiffrin E.L. (2001). Vascular remodeling in hypertension: Roles of apoptosis, inflammation, and fibrosis. Hypertension.

[25] Lan T.-H., Huang X.-Q., Tan H.-M. (2013). Vascular fibrosis in atherosclerosis. Cardiovasc. Pathol..

[26] Atkinson J. (2008). Age-related medial elastocalcinosis in arteries: Mechanisms, animal models, and physiological consequences. J. Appl. Physiol. (1985).

[27] Tsamis A., Krawiec J.T., Vorp D.A. (2013). Elastin and collagen fibre microstructure of the human aorta in ageing and disease: A review. J. R. Soc. Interface.

[28] Elliott R.J., McGrath L.T. (1994). Calcification of the human thoracic aorta during aging. Calcif. Tissue Int..

[29] Blumenthal H.T., Lansing A.I., Wheeler P.A. (1944). Calcification of the Media of the Human Aorta and Its Relation to Intimal Arteriosclerosis, Ageing and Disease. Am. J. Pathol..

[30] Lansing A.I., Alex M., Rosenthal T.B. (1950). Calcium and elastin in human arteriosclerosis. J. Gerontol..

[31] Zettervall S.L., Marshall A.P., Fleser P., Guzman R.J. (2018). Association of arterial calcification with chronic limb ischemia in patients with peripheral artery disease. J. Vasc. Surg..

[32] Chang Z., Yan H., Zhen Y., Zheng J., Liu Z. (2020). Lower Limb Arterial Calcification and Acute Thrombosis Risk in Patients with Peripheral Artery Disease. Ann. Vasc. Surg..

[33] Yan H., Chang Z., Liu Z. (2020). The risk factors for calcification vary among the different sections of the lower extremity artery in patients with symptomatic peripheral arterial disease. BMC Cardiovasc. Disord..

[34] Chen C., Wu Y., Lu H.-L., Liu K., Qin X. (2022). Identification of potential biomarkers of vascular calcification using bioinformatics analysis and validation in vivo. PeerJ.

[35] Lee S.J., Lee I.-K., Jeon J.-H. (2020). Vascular Calcification-New Insights Into Its Mechanism. Int. J. Mol. Sci..

[36] Taddei S., Virdis A., Ghiadoni L., Salvetti G., Bernini G., Magagna A., Salvetti A. (2001). Age-related reduction of NO availability and oxidative stress in humans. Hypertension.

[37] Martín-Fernández B., Gredilla R. (2016). Mitochondria and oxidative stress in heart aging. Age (Dordr).

[38] Trofor L. (2020). Evaluation of oxidative stress in smoking and non-smoking patients diagnosed with anxious-depressive disorder. Farmacia.

[39] Pagan L.U., Gomes M.J., Gatto M., Mota G.A.F., Okoshi K., Okoshi M.P. (2022). The Role of Oxidative Stress in the Aging Heart. Antioxidants.

[40] Martín-Fernández B., Gredilla R. (2018). Mitochondrial oxidative stress and cardiac ageing. Clin. Investig. Arterioscler..

[41] Veloso C.D., Belew G.D., Ferreira L.L., Grilo L.F., Jones J.G., Portincasa P., Sardão V.A., Oliveira P.J. (2019). A Mitochondrial Approach to Cardiovascular Risk and Disease. Curr. Pharm. Des..

[42] Csiszar A., Wang M., Lakatta E.G., Ungvari Z. (2008). Inflammation and endothelial dysfunction during aging: Role of NF-kappaB. J. Appl. Physiol. (1985).

[43] Qiao Y. (2022). Reactive Oxygen Species in Cardiovascular Calcification: Role of Medicinal Plants. Front. Pharmacol..

[44] Incalza M.A., D’Oria R., Natalicchio A., Perrini S., Laviola L., Giorgino F. (2018). Oxidative stress and reactive oxygen species in endothelial dysfunction associated with cardiovascular and metabolic diseases. Vascul. Pharmacol..

[45] Pfeifer M., Townsend R.R., Davies M.J., Vijapurkar U., Ren J. (2017). Effects of canagliflozin, a sodium glucose co-transporter 2 inhibitor, on blood pressure and markers of arterial stiffness in patients with type 2 diabetes mellitus: A post hoc analysis. Cardiovasc. Diabetol..

[46] Vlachopoulos C., Aznaouridis K., Stefanadis C. (2010). Prediction of cardiovascular events and all-cause mortality with arterial stiffness: A systematic review and meta-analysis. J. Am. Coll. Cardiol..

[47] Kodama S., Horikawa C., Fujihara K., Yoshizawa S., Yachi Y., Tanaka S., Ohara N., Matsunaga S., Yamada T., Hanyu O. (2014). Meta-analysis of the quantitative relation between pulse pressure and mean arterial pressure and cardiovascular risk in patients with diabetes mellitus. Am. J. Cardiol..

[48] Wei F.-F., Wu Y., Xue R., Liu X., He X., Dong B., Zhen Z., Chen X., Liang W., Zhao J. (2022). Clinical Significance of Mean and Pulse Pressure in Patients with Heart Failure with Preserved Ejection Fraction. Hypertension.

[49] Chow B., Rabkin S.W. (2015). The relationship between arterial stiffness and heart failure with preserved ejection fraction: A systemic meta-analysis. Heart Fail. Rev..

[50] Protogerou A.D., Vlachopoulos C., Thomas F., Zhang Y., Pannier B., Blacher J., Safar M.E. (2017). Longitudinal Changes in Mean and Pulse Pressure, and All-Cause Mortality: Data from 71,629 Untreated Normotensive Individuals. Am. J. Hypertens..

[51] Pitt B., Pfeffer M.A., Assmann S.F., Boineau R., Anand I.S., Claggett B., Clausell N., Desai A.S., Diaz R., Fleg J.L. (2014). Spironolactone for heart failure with preserved ejection fraction. N. Engl. J. Med..

[52] Liu L., Ni Y.-Q., Zhan J.-K., Liu Y.-S. (2021). The Role of SGLT2 Inhibitors in Vascular Aging. Aging Dis..

[53] Zhang X., Lim S.C., Tavintharan S., Yeoh L.Y., Sum C.F., Ang K., Yeo D., Low S., Kumari N. (2019). Association of central arterial stiffness with the presence and severity of diabetic retinopathy in Asians with type 2 diabetes. Diab. Vasc. Dis. Res..

[54] Yeboah K., Agyekum J.A., Owusu Mensah R.N.A., Affrim P.K., Adu-Gyamfi L., Doughan R.O., Adjei A.B. (2018). Arterial Stiffness Is Associated with Peripheral Sensory Neuropathy in Diabetes Patients in Ghana. J. Diabetes Res..

[55] Fu S., Guo Y., Luo L., Ye P. (2018). Association of arterial stiffness and central hemodynamics with moderately reduced glomerular filtration rate in Chinese middle-aged and elderly community residents: A cross-sectional analysis. BMC Nephrol..

[56] Chen L.-Q. (2014). SWEET sugar transporters for phloem transport and pathogen nutrition. New Phytol..

[57] Navale A.M., Paranjape A.N. (2016). Glucose transporters: Physiological and pathological roles. Biophys. Rev..

[58] Wasik A.A., Lehtonen S. (2018). Glucose Transporters in Diabetic Kidney Disease—Friends or Foes?. Front. Endocrinol..

[59] Liu B., Wang Y., Zhang Y., Yan B. (2019). Mechanisms of Protective Effects of SGLT2 Inhibitors in Cardiovascular Disease and Renal Dysfunction. Curr. Top. Med. Chem..

[60] Vallon V., Thomson S.C. (2017). Targeting renal glucose reabsorption to treat hyperglycaemia: The pleiotropic effects of SGLT2 inhibition. Diabetologia.

[61] Guthrie R.M. (2013). Sodium-glucose co-transporter 2 inhibitors and the potential for cardiovascular risk reduction in patients with type 2 diabetes mellitus. Postgrad. Med..

[62] Schubert M., Hansen S., Leefmann J., Guan K. (2020). Repurposing Antidiabetic Drugs for Cardiovascular Disease. Front. Physiol..

[63] Silva Dos Santos D., Polidoro J.Z., Borges-Júnior F.A., Girardi A.C.C. (2020). Cardioprotection conferred by sodium-glucose cotransporter 2 inhibitors: A renal proximal tubule perspective. Am. J. Physiol. Cell Physiol..

[64] Hodrea J., Saeed A., Molnar A., Fintha A., Barczi A., Wagner L.J., Szabo A.J., Fekete A., Balogh D.B. (2022). SGLT2 inhibitor dapagliflozin prevents atherosclerotic and cardiac complications in experimental type 1 diabetes. PLoS ONE.

[65] Haase V.H. (2006). Hypoxia-inducible factors in the kidney. Am. J. Physiol. Renal Physiol..

[66] Maruyama T., Takashima H., Oguma H., Nakamura Y., Ohno M., Utsunomiya K., Furukawa T., Tei R., Abe M. (2019). Canagliflozin Improves Erythropoiesis in Diabetes Patients with Anemia of Chronic Kidney Disease. Diabetes Technol. Ther..

[67] Lambers Heerspink H.J., de Zeeuw D., Wie L., Leslie B., List J. (2013). Dapagliflozin a glucose-regulating drug with diuretic properties in subjects with type 2 diabetes. Diabetes Obes. Metab..

[68] Sano M., Goto S. (2019). Possible Mechanism of Hematocrit Elevation by Sodium Glucose Cotransporter 2 Inhibitors and Associated Beneficial Renal and Cardiovascular Effects. Circulation.

[69] Lopaschuk G.D., Verma S. (2020). Mechanisms of Cardiovascular Benefits of Sodium Glucose Co-Transporter 2 (SGLT2) Inhibitors. JACC Basic Transl. Sci..

[70] Ehrenkranz J.R.L., Lewis N.G., Ronald Kahn C., Roth J. (2005). Phlorizin: A review. Diabetes Metab. Res. Rev..

[71] Peterson C. (1835). Analyse des Phloridzins.

[72] Kahn B.B., Shulman G.I., DeFronzo R.A., Cushman S.W., Rossetti L. (1991). Normalization of blood glucose in diabetic rats with phlorizin treatment reverses insulin-resistant glucose transport in adipose cells without restoring glucose transporter gene expression. J. Clin. Investig..

[73] Chasis H., Jolliffe N., Smith H.W. (1933). The action of phlorizin on the excretion of glucose, xylose, sucrose, creatinine and urea by man. J. Clin. Investig..

[74] Han J.H., Oh T.J., Lee G., Maeng H.J., Lee D.H., Kim K.M., Choi S.H., Jang H.C., Lee H.S., Park K.S. (2017). The beneficial effects of empagliflozin, an SGLT2 inhibitor, on atherosclerosis in ApoE−/− mice fed a western diet. Diabetologia.

[75] Shen L., You B.-A., Gao H.-Q., Li B.-Y., Yu F., Pei F. (2012). Effects of phlorizin on vascular complications in diabetes db/db mice. Chin. Med. J. (Engl.).

[76] Pei F., Li B.-Y., Zhang Z., Yu F., Li X.-L., Lu W., Cai Q., Gao H.-Q., Shen L. (2014). Beneficial effects of phlorizin on diabetic nephropathy in diabetic db/db mice. J. Diabetes Complicat..

[77] McGuire D.K., Shih W.J., Cosentino F., Charbonnel B., Cherney D.Z.I., Dagogo-Jack S., Pratley R., Greenberg M., Wang S., Huyck S. (2021). Association of SGLT2 Inhibitors with Cardiovascular and Kidney Outcomes in Patients with Type 2 Diabetes: A Meta-analysis. JAMA Cardiol..

[78] Scholten M., Midlöv P., Halling A. (2022). Disparities in prevalence of heart failure according to age, multimorbidity level and socioeconomic status in southern Sweden: A cross-sectional study. BMJ Open.

[79] Ziaeian B., Fonarow G.C. (2016). Epidemiology and aetiology of heart failure. Nat. Rev. Cardiol..

[80] Perry R.J., Shulman G.I. (2020). Sodium-glucose cotransporter-2 inhibitors: Understanding the mechanisms for therapeutic promise and persisting risks. J. Biol. Chem..

[81] Braunwald E. (2015). The war against heart failure: The Lancet lecture. Lancet.

[82] Mejhert M., Lindgren P., Schill O., Edner M., Persson H., Kahan T. (2013). Long term health care consumption and cost expenditure in systolic heart failure. Eur. J. Intern. Med..

[83] Zinman B., Wanner C., Lachin J.M., Fitchett D., Bluhmki E., Hantel S., Mattheus M., Devins T., Johansen O.E., Woerle H.J. (2015). Empagliflozin, Cardiovascular Outcomes, and Mortality in Type 2 Diabetes. N. Engl. J. Med..

[84] Neal B., Perkovic V., Mahaffey K.W., de Zeeuw D., Fulcher G., Erondu N., Shaw W., Law G., Desai M., Matthews D.R. (2017). Canagliflozin and Cardiovascular and Renal Events in Type 2 Diabetes. N. Engl. J. Med..

[85] Perkovic V., Jardine M.J., Neal B., Bompoint S., Heerspink H.J.L., Charytan D.M., Edwards R., Agarwal R., Bakris G., Bull S. (2019). Canagliflozin and Renal Outcomes in Type 2 Diabetes and Nephropathy. N. Engl. J. Med..

[86] Wiviott S.D., Raz I., Bonaca M.P., Mosenzon O., Kato E.T., Cahn A., Silverman M.G., Zelniker T.A., Kuder J.F., Murphy S.A. (2019). Dapagliflozin and Cardiovascular Outcomes in Type 2 Diabetes. N. Engl. J. Med..

[87] Brown E., Wilding J.P.H., Alam U., Barber T.M., Karalliedde J., Cuthbertson D.J. (2021). The expanding role of SGLT2 inhibitors beyond glucose-lowering to cardiorenal protection. Ann. Med..

[88] Scheen A.J. (2019). Cardiovascular and renal protection with sodium-glucose cotransporter type 2 inhibitors: New paradigm in type 2 diabetes management…and potentially beyond. Ann. Transl. Med..

[89] Lo K.B., Gul F., Ram P., Kluger A.Y., Tecson K.M., McCullough P.A., Rangaswami J. (2020). The Effects of SGLT2 Inhibitors on Cardiovascular and Renal Outcomes in Diabetic Patients: A Systematic Review and Meta-Analysis. Cardiorenal Med..

[90] Trum M., Riechel J., Wagner S. (2021). Cardioprotection by SGLT2 Inhibitors—Does It All Come Down to Na+?. Int. J. Mol. Sci..

[91] Baartscheer A., Schumacher C.A., Wüst R.C.I., Fiolet J.W.T., Stienen G.J.M., Coronel R., Zuurbier C.J. (2017). Empagliflozin decreases myocardial cytoplasmic Na^+^ through inhibition of the cardiac Na^+^/H^+^ exchanger in rats and rabbits. Diabetologia.

[92] Uthman L., Homayr A., Juni R.P., Spin E.L., Kerindongo R., Boomsma M., Hollmann M.W., Preckel B., Koolwijk P., van Hinsbergh V.W.M. (2019). Empagliflozin and Dapagliflozin Reduce ROS Generation and Restore NO Bioavailability in Tumor Necrosis Factor α-Stimulated Human Coronary Arterial Endothelial Cells. Cell Physiol. Biochem..

[93] Trum M., Riechel J., Lebek S., Pabel S., Sossalla S.T., Hirt S., Arzt M., Maier L.S., Wagner S. (2020). Empagliflozin inhibits Na+ /H+ exchanger activity in human atrial cardiomyocytes. ESC Heart Fail..

[94] Trum M., Wagner S., Maier L.S., Mustroph J. (2020). CaMKII and GLUT1 in heart failure and the role of gliflozins. Biochim. Biophys. Acta Mol. Basis Dis..

[95] Philippaert K., Kalyaanamoorthy S., Fatehi M., Long W., Soni S., Byrne N.J., Barr A., Singh J., Wong J., Palechuk T. (2021). Cardiac Late Sodium Channel Current Is a Molecular Target for the Sodium/Glucose Cotransporter 2 Inhibitor Empagliflozin. Circulation.

[96] Hou Y.-C., Zheng C.-M., Yen T.-H., Lu K.-C. (2020). Molecular Mechanisms of SGLT2 Inhibitor on Cardiorenal Protection. Int. J. Mol. Sci..

[97] He X., Du T., Long T., Liao X., Dong Y., Huang Z.-P. (2022). Signaling cascades in the failing heart and emerging therapeutic strategies. Signal Transduct. Target. Ther..

[98] Sadoshima J., Izumo S. (1997). The cellular and molecular response of cardiac myocytes to mechanical stress. Annu. Rev. Physiol..

[99] Verma S., Jüni P., Mazer C.D. (2019). Pump, pipes, and filter: Do SGLT2 inhibitors cover it all?. Lancet.

[100] Verma S., McMurray J.J.V. (2018). SGLT2 inhibitors and mechanisms of cardiovascular benefit: A state-of-the-art review. Diabetologia.

[101] Zelniker T.A., Wiviott S.D., Raz I., Im K., Goodrich E.L., Bonaca M.P., Mosenzon O., Kato E.T., Cahn A., Furtado R.H.M. (2019). SGLT2 inhibitors for primary and secondary prevention of cardiovascular and renal outcomes in type 2 diabetes: A systematic review and meta-analysis of cardiovascular outcome trials. Lancet.

[102] Khunti K. (2021). SGLT2 inhibitors in people with and without T2DM. Nat. Rev. Endocrinol..

[103] Boutagy N.E., Singh A.K., Sessa W.C. (2022). Targeting the vasculature in cardiometabolic disease. J. Clin. Investig..

[104] Pi X., Xie L., Patterson C. (2018). Emerging Roles of Vascular Endothelium in Metabolic Homeostasis. Circ. Res..

[105] Hasan S.S., Fischer A. (2021). The Endothelium: An Active Regulator of Lipid and Glucose Homeostasis. Trends Cell Biol..

[106] Fadini G.P. (2020). SGLT-2 Inhibitors and Circulating Progenitor Cells in Diabetes. Cell Metab..

[107] Hess D.A., Terenzi D.C., Trac J.Z., Quan A., Mason T., Al-Omran M., Bhatt D.L., Dhingra N., Rotstein O.D., Leiter L.A. (2019). SGLT2 Inhibition with Empagliflozin Increases Circulating Provascular Progenitor Cells in People with Type 2 Diabetes Mellitus. Cell Metab..

[108] Hess D.A., Terenzi D.C., Verma S. (2020). SGLT-2 Inhibitors and Regenerative Cell Exhaustion. Cell Metab..

[109] Monami M., Dicembrini I., Mannucci E. (2017). Effects of SGLT-2 inhibitors on mortality and cardiovascular events: A comprehensive meta-analysis of randomized controlled trials. Acta Diabetol..

[110] Benham J.L., Booth J.E., Sigal R.J., Daskalopoulou S.S., Leung A.A., Rabi D.M. (2021). Systematic review and meta-analysis: SGLT2 inhibitors, blood pressure and cardiovascular outcomes. Int. J. Cardiol. Heart Vasc..

[111] Chilton R., Tikkanen I., Cannon C.P., Crowe S., Woerle H.J., Broedl U.C., Johansen O.E. (2015). Effects of empagliflozin on blood pressure and markers of arterial stiffness and vascular resistance in patients with type 2 diabetes. Diabetes Obes. Metab..

[112] Davies M.J., Merton K., Vijapurkar U., Yee J., Qiu R. (2017). Efficacy and safety of canagliflozin in patients with type 2 diabetes based on history of cardiovascular disease or cardiovascular risk factors: A post hoc analysis of pooled data. Cardiovasc. Diabetol..

[113] Ye Y., Bajaj M., Yang H.-C., Perez-Polo J.R., Birnbaum Y. (2017). SGLT-2 Inhibition with Dapagliflozin Reduces the Activation of the Nlrp3/ASC Inflammasome and Attenuates the Development of Diabetic Cardiomyopathy in Mice with Type 2 Diabetes. Further Augmentation of the Effects with Saxagliptin, a DPP4 Inhibitor. Cardiovasc. Drugs Ther..

[114] Gaspari T., Spizzo I., Liu H., Hu Y., Simpson R.W., Widdop R.E., Dear A.E. (2018). Dapagliflozin attenuates human vascular endothelial cell activation and induces vasorelaxation: A potential mechanism for inhibition of atherogenesis. Diabetes Vasc. Dis. Res..

[115] Lee T.-M., Chang N.-C., Lin S.-Z. (2017). Dapagliflozin, a selective SGLT2 Inhibitor, attenuated cardiac fibrosis by regulating the macrophage polarization via STAT3 signaling in infarcted rat hearts. Free Radic. Biol. Med..

[116] Li H., Shin S.E., Seo M.S., An J.R., Choi I.-W., Jung W.-K., Firth A.L., Lee D.-S., Yim M.-J., Choi G. (2018). The anti-diabetic drug dapagliflozin induces vasodilation via activation of PKG and Kv channels. Life Sci..

[117] Aragón-Herrera A., Feijóo-Bandín S., Otero Santiago M., Barral L., Campos-Toimil M., Gil-Longo J., Costa Pereira T.M., García-Caballero T., Rodríguez-Segade S., Rodríguez J. (2019). Empagliflozin reduces the levels of CD36 and cardiotoxic lipids while improving autophagy in the hearts of Zucker diabetic fatty rats. Biochem. Pharmacol..

[118] Birnbaum Y., Bajaj M., Yang H.C., Ye Y. (2018). Combined SGLT2 and DPP4 Inhibition Reduces the Activation of the Nlrp3/ASC Inflammasome and Attenuates the Development of Diabetic Nephropathy in Mice with Type 2 Diabetes. Cardiovasc. Drugs Ther..

[119] Andreadou I., Efentakis P., Balafas E., Togliatto G., Davos C.H., Varela A., Dimitriou C.A., Nikolaou P.-E., Maratou E., Lambadiari V. (2017). Empagliflozin Limits Myocardial Infarction in Vivo and Cell Death in Vitro: Role of STAT3, Mitochondria, and Redox Aspects. Front. Physiol..

[120] Lin B., Koibuchi N., Hasegawa Y., Sueta D., Toyama K., Uekawa K., Ma M., Nakagawa T., Kusaka H., Kim-Mitsuyama S. (2014). Glycemic control with empagliflozin, a novel selective SGLT2 inhibitor, ameliorates cardiovascular injury and cognitive dysfunction in obese and type 2 diabetic mice. Cardiovasc. Diabetol..

[121] Zhou H., Wang S., Zhu P., Hu S., Chen Y., Ren J. (2018). Empagliflozin rescues diabetic myocardial microvascular injury via AMPK-mediated inhibition of mitochondrial fission. Redox Biol..

[122] Quintero M., Colombo S.L., Godfrey A., Moncada S. (2006). Mitochondria as signaling organelles in the vascular endothelium. Proc. Natl. Acad. Sci. USA.

[123] Fuhrmann D.C., Brüne B. (2017). Mitochondrial composition and function under the control of hypoxia. Redox Biol..

[124] Aroor A.R., Das N.A., Carpenter A.J., Habibi J., Jia G., Ramirez-Perez F.I., Martinez-Lemus L., Manrique-Acevedo C.M., Hayden M.R., Duta C. (2018). Glycemic control by the SGLT2 inhibitor empagliflozin decreases aortic stiffness, renal resistivity index and kidney injury. Cardiovasc. Diabetol..

[125] Calabia J., Torguet P., Garcia I., Martin N., Mate G., Marin A., Molina C., Valles M. (2014). The relationship between renal resistive index, arterial stiffness, and atherosclerotic burden: The link between macrocirculation and microcirculation. J. Clin. Hypertens. (Greenwich).

[126] Mitchell G.F. (2008). Effects of central arterial aging on the structure and function of the peripheral vasculature: Implications for end-organ damage. J. Appl. Physiol. (1985).

[127] Behnammanesh G., Durante G.L., Khanna Y.P., Peyton K.J., Durante W. (2020). Canagliflozin inhibits vascular smooth muscle cell proliferation and migration: Role of heme oxygenase-1. Redox Biol..

[128] Durante W., Behnammanesh G., Peyton K.J. (2021). Effects of Sodium-Glucose Co-Transporter 2 Inhibitors on Vascular Cell Function and Arterial Remodeling. Int. J. Mol. Sci..

[129] Heerspink H.J.L., Perco P., Mulder S., Leierer J., Hansen M.K., Heinzel A., Mayer G. (2019). Canagliflozin reduces inflammation and fibrosis biomarkers: A potential mechanism of action for beneficial effects of SGLT2 inhibitors in diabetic kidney disease. Diabetologia.

[130] Garvey W.T., Van Gaal L., Leiter L.A., Vijapurkar U., List J., Cuddihy R., Ren J., Davies M.J. (2018). Effects of canagliflozin versus glimepiride on adipokines and inflammatory biomarkers in type 2 diabetes. Metabolism.

[131] Mancini S.J., Boyd D., Katwan O.J., Strembitska A., Almabrouk T.A., Kennedy S., Palmer T.M., Salt I.P. (2018). Canagliflozin inhibits interleukin-1β-stimulated cytokine and chemokine secretion in vascular endothelial cells by AMP-activated protein kinase-dependent and -independent mechanisms. Sci. Rep..

[132] Day E.A., Ford R.J., Lu J.H., Lu R., Lundenberg L., Desjardins E.M., Green A.E., Lally J.S.V., Schertzer J.D., Steinberg G.R. (2020). The SGLT2 inhibitor canagliflozin suppresses lipid synthesis and interleukin-1 beta in ApoE deficient mice. Biochem. J..

[133] Cai C., Guo Z., Chang X., Li Z., Wu F., He J., Cao T., Wang K., Shi N., Zhou H. (2022). Empagliflozin attenuates cardiac microvascular ischemia/reperfusion through activating the AMPKα1/ULK1/FUNDC1/mitophagy pathway. Redox Biol..

[134] Wu W., Tian W., Hu Z., Chen G., Huang L., Li W., Zhang X., Xue P., Zhou C., Liu L. (2014). ULK1 translocates to mitochondria and phosphorylates FUNDC1 to regulate mitophagy. EMBO Rep..

[135] Tian W., Li W., Chen Y., Yan Z., Huang X., Zhuang H., Zhong W., Chen Y., Wu W., Lin C. (2015). Phosphorylation of ULK1 by AMPK regulates translocation of ULK1 to mitochondria and mitophagy. FEBS Lett..

[136] Li G., Li J., Shao R., Zhao J., Chen M. (2021). FUNDC1: A Promising Mitophagy Regulator at the Mitochondria-Associated Membrane for Cardiovascular Diseases. Front. Cell Dev. Biol..

[137] Juni R.P., Kuster D.W.D., Goebel M., Helmes M., Musters R.J.P., van der Velden J., Koolwijk P., Paulus W.J., van Hinsbergh V.W.M. (2019). Cardiac Microvascular Endothelial Enhancement of Cardiomyocyte Function Is Impaired by Inflammation and Restored by Empagliflozin. JACC Basic Transl. Sci..

[138] Mantsounga C.S., Morrison A.R. (2019). Empagliflozin and Protecting Microvascular Support of Heart Mechanics. JACC Basic Transl. Sci..

[139] Soares R.N., Ramirez-Perez F.I., Cabral-Amador F.J., Morales-Quinones M., Foote C.A., Ghiarone T., Sharma N., Power G., Smith J.A., Rector R.S. (2022). SGLT2 Inhibition Attenuates Arterial Dysfunction and Decreases Vascular F-Actin Content and Expression of Proteins Associated with Oxidative Stress in Aged Mice. Geroscience.

[140] Salim H.M., Fukuda D., Yagi S., Soeki T., Shimabukuro M., Sata M. (2016). Glycemic Control with Ipragliflozin, a Novel Selective SGLT2 Inhibitor, Ameliorated Endothelial Dysfunction in Streptozotocin-Induced Diabetic Mouse. Front. Cardiovasc. Med..

[141] Hidalgo Santiago J.C., Maraver Delgado J., Cayón Blanco M., López Saez J.B., Gómez-Fernández P. (2020). Effect of dapagliflozin on arterial stiffness in patients with type 2 diabetes mellitus. Med. Clin. (Barc.).

[142] Bosch A., Ott C., Jung S., Striepe K., Karg M.V., Kannenkeril D., Dienemann T., Schmieder R.E. (2019). How does empagliflozin improve arterial stiffness in patients with type 2 diabetes mellitus? Sub analysis of a clinical trial. Cardiovasc. Diabetol..

[143] Jung S., Bosch A., Kannenkeril D., Karg M.V., Striepe K., Bramlage P., Ott C., Schmieder R.E. (2020). Combination of empagliflozin and linagliptin improves blood pressure and vascular function in type 2 diabetes. Eur. Heart J. Cardiovasc. Pharmacother..

[144] Bekki M., Tahara N., Tahara A., Igata S., Honda A., Sugiyama Y., Nakamura T., Sun J., Kumashiro Y., Matsui T. (2019). Switching Dipeptidyl Peptidase-4 Inhibitors to Tofogliflozin, a Selective Inhibitor of Sodium-Glucose Cotransporter 2 Improve Arterial Stiffness Evaluated by Cardio-Ankle Vascular Index in Patients with Type 2 Diabetes: A Pilot Study. Curr. Vasc. Pharmacol..

[145] Katakami N., Mita T., Yoshii H., Shiraiwa T., Yasuda T., Okada Y., Torimoto K., Umayahara Y., Kaneto H., Osonoi T. (2021). Effect of tofogliflozin on arterial stiffness in patients with type 2 diabetes: Prespecified sub-analysis of the prospective, randomized, open-label, parallel-group comparative UTOPIA trial. Cardiovasc. Diabetol..

[146] Katakami N., Mita T., Yoshii H., Shiraiwa T., Yasuda T., Okada Y., Torimoto K., Umayahara Y., Kaneto H., Osonoi T. (2020). Tofogliflozin does not delay progression of carotid atherosclerosis in patients with type 2 diabetes: A prospective, randomized, open-label, parallel-group comparative study. Cardiovasc. Diabetol..

[147] Groenewegen K., den Ruijter H., Pasterkamp G., Polak J., Bots M., Peters S.A. (2016). Vascular age to determine cardiovascular disease risk: A systematic review of its concepts, definitions, and clinical applications. Eur. J. Prev. Cardiol..

[148] Bruno R.M., Nilsson P.M., Engström G., Wadström B.N., Empana J.-P., Boutouyrie P., Laurent S. (2020). Early and Supernormal Vascular Aging. Hypertension.

[149] Nilsson P.M., Boutouyrie P., Laurent S. (2009). Vascular Aging: A Tale of EVA and ADAM in Cardiovascular Risk Assessment and Prevention. Hypertension.

[150] Olsen M.H., Angell S.Y., Asma S., Boutouyrie P., Burger D., Chirinos J.A., Damasceno A., Delles C., Gimenez-Roqueplo A.-P., Hering D. (2016). A call to action and a lifecourse strategy to address the global burden of raised blood pressure on current and future generations: The Lancet Commission on hypertension. Lancet.

[151] Laurent S., Boutouyrie P., Cunha P.G., Lacolley P., Nilsson P.M. (2019). Concept of Extremes in Vascular Aging: From Early Vascular Aging to Supernormal Vascular Aging. Hypertension.

[152] Lemogoum D., Ngatchou W., Janssen C., Leeman M., Van Bortel L., Boutouyrie P., Degaute J.P., Van de Borne P. (2012). Effects of Hunter-Gatherer Subsistence Mode on Arterial Distensibility in Cameroonian Pygmies. Hypertension.

[153] Kaplan H., Thompson R.C., Trumble B.C., Wann L.S., Allam A.H., Beheim B., Frohlich B., Sutherland M.L., Sutherland J.D., Stieglitz J. (2017). Coronary atherosclerosis in indigenous South American Tsimane: A cross-sectional cohort study. Lancet.

[154] Gurven M., Blackwell A.D., Rodríguez D.E., Stieglitz J., Kaplan H. (2012). Does Blood Pressure Inevitably Rise With Age?: Longitudinal Evidence Among Forager-Horticulturalists. Hypertension.

[155] Niiranen T.J., Lyass A., Larson M.G., Hamburg N.M., Benjamin E.J., Mitchell G.F., Vasan R.S. (2017). Prevalence, Correlates, and Prognosis of Healthy Vascular Aging in a Western Community-Dwelling Cohort: The Framingham Heart Study. Hypertension.

[156] (2010). Reference Values for Arterial Stiffness’ Collaboration Determinants of pulse wave velocity in healthy people and in the presence of cardiovascular risk factors: “Establishing normal and reference values”. Eur. Heart J..

[157] Wilkinson I.B., Franklin S.S., Cockcroft J.R. (2004). Nitric Oxide and the Regulation of Large Artery Stiffness: From Physiology to Pharmacology. Hypertension.

[158] Celermajer D.S., Sorensen K.E., Spiegelhalter D.J., Georgakopoulos D., Robinson J., Deanfield J.E. (1994). Aging is associated with endothelial dysfunction in healthy men years before the age-related decline in women. J. Am. Coll. Cardiol..

[159] Barbu E., Popescu M.-R., Popescu A.-C., Balanescu S.-M. (2022). Inflammation as A Precursor of Atherothrombosis, Diabetes and Early Vascular Aging. Int. J. Mol. Sci..

[160] Bovolini A., Garcia J., Andrade M.A., Duarte J.A. (2021). Metabolic Syndrome Pathophysiology and Predisposing Factors. Int. J. Sports Med..

[161] Prattichizzo F., De Nigris V., La Sala L., Procopio A.D., Olivieri F., Ceriello A. (2016). “Inflammaging” as a Druggable Target: A Senescence-Associated Secretory Phenotype-Centered View of Type 2 Diabetes. Oxid. Med. Cell. Longev..

[162] Prattichizzo F., De Nigris V., Spiga R., Mancuso E., La Sala L., Antonicelli R., Testa R., Procopio A.D., Olivieri F., Ceriello A. (2018). Inflammageing and metaflammation: The yin and yang of type 2 diabetes. Ageing Res. Rev..

[163] Kuryłowicz A., Koźniewski K. (2020). Anti-Inflammatory Strategies Targeting Metaflammation in Type 2 Diabetes. Molecules.

[164] Paisley A.N., Paisley A.J., Yadav R., Younis N., Rao-Balakrishna P., Soran H. (2013). Dapagliflozin: A review on efficacy, clinical effectiveness and safety. Expert Opin. Investig. Drugs.

[165] El Assar M., Angulo J., Rodríguez-Mañas L. (2013). Oxidative stress and vascular inflammation in aging. Free Radic. Biol. Med..

